# Deal with the Druggist!

**Published:** 1859-02

**Authors:** 


### *Deal with the Druggist!*

Country physicians often procure their medicines from merchants in their neighborhoods, or they send their orders for medicines, instruments, etc., by these merchants, who from ignorance of the standing of dealers in drugs, or from a desire to obtain their supplies at the lowest possible prices, deal with second or third rate establishments.

Now, this is a very grave error. All drug dealers are not druggists. Medicines *can* be bought at some large establishment in this and other cities at lower rates than at others; but when this is done, it is always at the sacrifice of purity, and the physician who buys such medicines, is in great danger of compromising his standing as a reliable practitioner, by employing remedies which are deprived by adulterations of their normal power. This is a very serious mattter, and the physician should always take care that his shelves and drawers are supplied with an assortment of medicines procurred *from a reliable source*, even if they should cost him a little more than if procured by his merchant who deals with the seller of cheap, (and consequently adulterated or damaged) drugs. He will be more than repaid for the small extra outlay, by the certainty of the action of his remedies, and the consequent reliability of his practice.

Our subscribers would consult their own interest by informing themselves in regard to the reliability of those from whom their medicines are procured. We would advise them by all means to *deal with the competent druggist*, and not with the mere vender of drugs!—*Medical & Surgical Reporter.*

